# Radiological progression of medulloblastoma from non-existence to symptomatic mass in 42 days: Case report, kinetic analysis, and literature review

**DOI:** 10.1016/j.bas.2025.104188

**Published:** 2025-01-18

**Authors:** Yousef M. Odeibat, Mohammad Y. Hiasat, Mohammad H.M. Alhazaimeh, Ala Marji, Ammar S. Al-Omary, Ajwad Obeidat, Amer A. Alomari

**Affiliations:** a Neuron Clinics, Amman, Jordan; b Division of Neurosurgery, Department of Clinical Sciences, Faculty of Medicine, Yarmouk University, Irbid, Jordan; c KHCC, Jordan; d Advanced skullbase & neuro-oncology fellowship, San Filippo Neri, Italy; e Ministry of Health, Irbid, Jordan; f Division of Neurosurgery, Department of Special Surgery, Faculty of Medicine, Mutah University, Al-Karak, Jordan

**Keywords:** Medulloblastoma, Doubling time, Growth rate, Kinetic analysis, Pediatric, Neuro-oncology

## Abstract

**Introduction:**

Medulloblastoma (MB) is the most common malignant brain tumor in children, characterized by its aggressive nature and rapid growth. However, the standard practice of immediate surgical intervention limits opportunities to study the tumor's doubling time (DT), thereby restricting our understanding of its growth kinetics.

**Research question:**

This study presents a pediatric case of MB that developed within 42 days of a normal MRI, with a focus on analyzing the tumor's DT and comparing it with values reported in the literature.

**Materials and methods:**

Tumor kinetics were assessed using volumetric measurements obtained via 3D Slicer software. A literature review identified six studies that examined the radiological DT of medulloblastoma for comparative analysis.

**Results:**

The estimated DT for the presented case ranged between 2 and 6.5 days, indicating exceptionally rapid tumor proliferation. This is shorter than previously reported cases, where DTs ranged from 6.84 to 353 days. Including the current case, the mean DT across all cases is 53.8 days.

**Discussion and conclusion:**

This case highlights the potential for extremely rapid progression in MB, underscoring the aggressive nature of this tumor. The findings emphasize the importance of prompt diagnosis and intervention in MB cases. Further research into the genetic and molecular mechanisms driving its aggressive growth could support the development of targeted therapeutic strategies.

## Highlights

• Doubling time measures how long it takes for a tumor to double in size.
• This case reports the shortest medulloblastoma doubling time documented in the literature.
• A literature review reveals that medulloblastomas exhibit variable doubling times.
• Understanding the factors contributing to this variability may help guide targeted treatments.

## 1 Introduction

Medulloblastoma (MB) is a rapidly growing WHO grade IV embryonal brain tumor (Cushing, 1930; Torp et al., 2022). It is the most common malignant pediatric brain tumor, accounting for approximately 50% of posterior fossa tumors (Orr, 2020). Surgical intervention of these tumors is a standard practice upon detection, resulting in a short interval from symptom onset to surgical excision. Thus, while doubling time (DT) is an important kinetic measurement to appreciate the aggressiveness of brain tumors (Ambica and Anupama, 2021; Blankenberg et al., 1995), there is limited opportunity to study DT in MB (Perret et al., 2011; Bacon et al., 2005).

In this article, we present a case that offers a unique opportunity to investigate the kinetics of MB in pediatrics. Our patient is an infant who demonstrated a radiologically documented progression from a previously negative brain magnetic resonance image (MRI) to a 5.9 cm³ MB within 42 days. Then, we performed a volumetric and kinetic analysis to estimate the growth rate and DT of the tumor, aligning our findings with existing literature. To our knowledge, this case represents the fastest radiologically documented progression of MB in the literature.

## 2 Case report

A male born to non-consanguineous parents via In Vitro Fertilization (IVF) was delivered prematurely by cesarean section at 32 weeks gestational age. Following birth, the patient was admitted to the Neonatal Intensive Care Unit (NICU) for 15 days, requiring surfactant treatment and mechanical ventilation. During this time, the patient developed a chest infection and sepsis, for which antibiotic therapy was administered. After being discharged, the patient was later readmitted for surgical management of a left hip effusion.

Since birth, the patient has demonstrated weakness in the right upper limb, which was diagnosed as a birth-related brachial plexus injury. At a subsequent follow-up, a pediatric neurologist requested a non-contrast brain magnetic resonance imaging (MRI), performed at the age of 139 days. The MRI reported no abnormalities.

Subsequently, the patient was brought to our attention at 181 days old (42 days after the previous normal brain MRI) with symptoms of marked irritability, feeding difficulties, and recurrent vomiting. On examination, the anterior fontanelle was full and tense. An emergent non-contrast brain computed tomography (CT) scan was performed, revealing the presence of a posterior fossa irregular, cystic, and lobulated heterogeneous lesion. It compressed the fourth ventricle and extended through the right foramen of Luschka, resulting in obstructive triventricular acute hydrocephalus. Consequently, the patient underwent urgent surgery for insertion of a right frontal ventriculoperitoneal shunt.

**Fig. 1:**
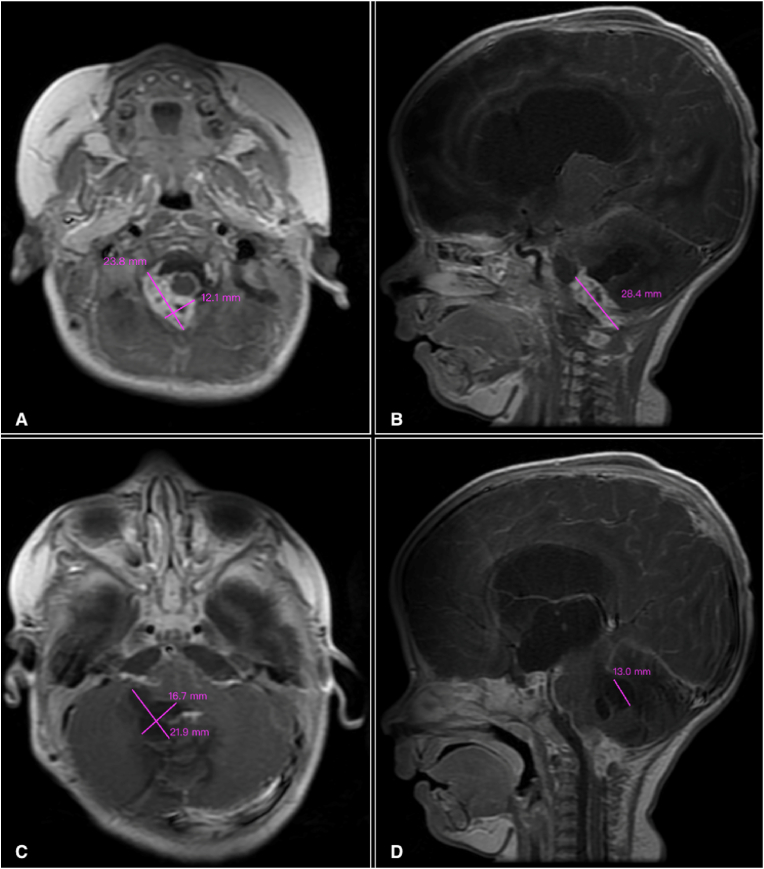
Contrasted brain MRI at 182 days old. (A) Axial view demonstrating heterogeneously enhancing solid mass occupying the right cerebellopontine angle (CPA). (B) Sagittal view demonstrates the mass in the right CPA, the cystic intracerebellar component, and the dilated lateral ventricles. (C) Axial view demonstrating cerebellar multilobulated cystic component. (D) Sagittal view demonstrates the cystic component, the solid component extending through the foramen magnum, and the dilated third and lateral ventricles.

On the next day, at the age of 182 days, the patient had contrasted brain MRI (Fig. 1A–D) which showed a heterogeneously enhancing solid mass in the right foramen of Luschka, extending laterally into the right cerebellopontine angle (CPA), then downward to occupy the right side of the cisterna magna and continues through the foramen magnum to the level of C1/C2, with dimensions measuring 2.38 × 1.21 × 2.84 cm. At the masse's left superior margin, there is a cerebellar lobulated cystic mass measuring 2.19 × 1.67 × 1.3 cm, compressing the fourth ventricle.

Eventually, the patient underwent craniotomy and excision of the posterior fossa mass. Histopathological analysis suggested an embryonal tumor, probably MB, characterized by a diffuse growth pattern. Microscopic examination revealed hyperchromatic, small, round nuclei with overlapping and inconspicuous nucleoli. Immunohistochemically, the tumor cells tested positive for synaptophysin and chromogranin and exhibited focal positivity for GFAP and vimentin. H3K27me3 was mutated, while H3K27M was non-mutant. Over 10% of the tumor cells expressed p53. The tumor lacked mutations in INI1 and BRG1, and was negative for OLIG2, S100, c-MYC, EMA, NeuN, IDH-1 (R132H), CD43, CD45, and CD68. The Ki-67 labeling index (LI) was notably high, reaching 30%.

## 3 Kinetic analysis

The exponential growth of tumor volume can be described by the following equations and definitions (Talkington and Durrett, 2015).

1. Exponential Growth Model:

(1) $$V\left(t\right)={V_{0}e}^{rt}$$

This equation models the tumor volume V(t) at time t, where $V_{0}$ is the initial tumor volume, and r is the growth rate.

2. Derivation of Growth Rate:

Starting from the exponential growth model, the growth rate can be derived as follows:

(2) $$r=\frac{\ln\left(\frac{V\left(t\right)}{V_{0}}\right)}{t}$$

By taking the natural logarithm of both sides of Equation (1) and solving for r, we obtain this expression, which allows calculating the growth rate based on the known values of V(t), $V_{0}$, and t.

3. Doubling Time Calculation:

(3) $$DT=\frac{\ln\left(2\right)}{r}$$

Doubling time (DT) is the time required for the tumor volume to double, which is inversely proportional to the growth rate.

In our case, no tumor was identified in the MRI done at age 139 days, thus $V_{0}=0$. Applying this value in Equation (2) makes the formula invalid due to division by zero. Yet, it is noteworthy to measure the DT of our case. Thus, it is estimated using the following assumptions based on the MRI done at age 139 days:

1. There is a tumor which is undetectable on MRI due to its small size.
2. The minimum possible initial diameter is 150 μm, approximating the diameter of a single medulloblastoma stem cell (Gong et al., 2018).
3. The maximum estimated diameter of a mass not identifiable on this MRI is 4.9 mm, given the slice thickness is 5 mm.
4. Assumptions 2 and 3 can help to estimate a range for the doubling time.
5. If the tumor emerged after the MRI, the DT would be even shorter than the calculated estimates.

**Fig. 2:**
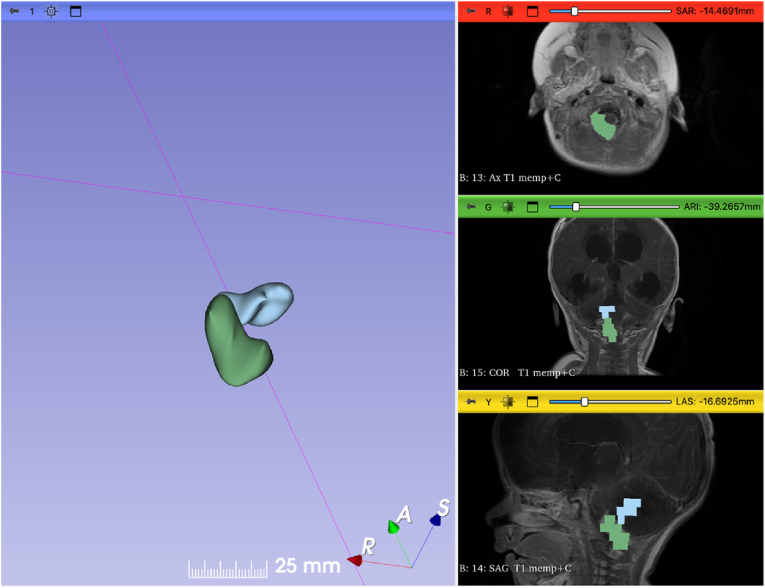
Snapshot from 3D Slicer software showing a 3D model of the tumor created using the Segment Editor module.

For the volumetric analysis of the tumor, we utilized 3D Slicer 5.6.2 (Fedorov et al., 2012), an open-source software platform for medical image informatics, image processing, and three-dimensional visualization (http://www.slicer.org). The Segment Editor module was used to create a 3D model of the tumor. Volume measurements were obtained through the Segment Statistics module's segment labelmap statistics. The tumor's solid and cystic components were measured separately using the MRI done at age 182 days, with volumes of 3.6 cm³ and 2.3 cm³, respectively (Fig. 2). Based on these measurements and the aforementioned assumptions and equations, the DT of the tumor was estimated to be in the range of 2–6.5 days (Table 1).

**Table 1:** Estimated doubling time of the tumor based on initial diameter assumptions. Given that t=43days, V(t)=5.9cm^3^.

Assumptions	Initial Diameter	Initial Volume (cm³)	Growth Rate (per day)	Doubling Time (days)
Assumption 1	150 μm	1.767 × 10^−6^	0.349	2
Assumption 2	4.9 mm	6.16 × 10^−2^	0.106	6.5

## 4 Discussion

MB primarily affects children but can also occur in adults. It is the most common malignant brain tumor in the pediatric population, accounting for approximately 20% of all pediatric brain tumors in the first decade of their life (Alcantara et al., 2023). MB is an aggressive grade IV tumor (Torp et al., 2022), and the duration from symptoms onset to diagnosis is typically short. Surgical intervention for posterior fossa tumors is a standard practice upon detection. Consequently, there is limited opportunity to study the natural growth of this tumor (Perret et al., 2011; Bacon et al., 2005).

The reported case demonstrates a pediatric MB that progressed radiologically from non-existence to a symptomatic mass within 42 days since the last negative brain MRI, with an MRI on day 43 revealing a tumor measuring 5.9 cm³. Quantifying the DT in the presented case provides an opportunity to put our findings in parallel with other cases in the literature. We made assumptions regarding the tumor's initial volume to make our calculations mathematically possible. We assumed that there was an undetectable tumor when the negative MRI was done at age 139 days and hypothesized that its diameter would fall between 150 μm and 4.9 mm. Accordingly, we could calculate the estimated DT, which ranged between 2 and 6.5 days. We found our method of assuming an initial volume similar to that previously demonstrated by Yamashita et al. (Yamashita and Kuwabara, 1983) (Table 2 – case 3).

**Table 2:** Literature review of radiological doubling time in medulloblastoma.

Reference	Case	Age	Sex	$V_{0}$ in cm^3^	V(t) in cm^3^	t in days	DT in days	DT equation	LI	Primary vs. Recurrent
Yamashita et al., 1983	1	12 yr	M	0.7	32.5	109	19.4	$DT=\frac{\log\left(2\right)}{\log\left(\frac{V\left(t\right)}{V_{0}}\right)}\;\cdot t$	Not reported	Primary
	2	12 yr	M	4.7	22.3	95	20.9		Not reported	Primary
	3	5 yr	F	N/A^a^	5.6	495	19.2		Not reported	Primary
Hirakawa et al., 1986	4	5 yr	M	1.6 × 10^−2^	7.9	593	69	$DT=\frac{\ln\left(2\right)}{\ln\left(\frac{V\left(t\right)}{V_{0}}\right)}\;\cdot t$	^3^H-thymidine LI = 14%	Primary
	5	4 mo	M	3.3 × 10^−2^	4.2	65	9.4		^3^H-thymidine LI = 14%	Primary
Ito et al., 1992	6	32 yr	M	5.9	17.1	32	20.7	$DT=\frac{\log\left(2\right)}{\log\left(\frac{V\left(t\right)}{V_{0}}\right)}\;\cdot t$	BUdR LI = 8.0 ± 0.9%	Primary
	7	10 yr	M	5.2	66.8	90	24.4		BUdR LI = 9.7 ± 1.3%	Recurrent
	8	21 yr	M	1.1	22.4	105	23.8		BUdR LI = 19.7 ± 1.3%	Recurrent
Bacon et al., 2005	9	10 yr	M	3.88	32.55	21	6.84	$DT=\frac{\log\left(2\right)}{\log\left(\frac{V\left(t\right)}{V_{0}}\right)}\;\cdot t$	Not reported	Recurrent
Doron et al., 2016	10	4 yr	M	4.18 × 10^−3^	18.816	946	78	$DT=\frac{\ln\left(2\right)}{\ln\left(\frac{V\left(t\right)}{V_{0}}\right)}\;\cdot t$	MIB-I LI = 35–40%	Primary
Golpayegani et al., 2018^b^	11_a_	4 yr	M	<1	4.2	731	353	$DT=\frac{\log\left(2\right)}{\log\left(\frac{V\left(t\right)}{V_{0}}\right)}\;\cdot t$	Not reported	Primary
	11_b_			4.2	9.5	60	50.9		Not reported	Primary
Present case	12	6 mo	M	N/A	5.9	43	[2–6.5]	$DT=\frac{\ln\left(2\right)}{\ln\left(\frac{V\left(t\right)}{V_{0}}\right)}\;\cdot t$	Ki-67 LI = 30%	Primary

Vo: Measured initial tumor volume. V(t): final tumor volume at time t. t: interval between initial and final volumes. DT: doubling time. y: year-old. mo: month-old. d: day-old. M: male. F: female. BudR: bromodeoxyuridine. LI: labeling index.

a The initial tumor volume was hypothesized by the author based on the belief that a tumor was present but not detectable on the last negative CT scan. It is hypothesized that the initial tumor consists of 10^8^ cells. The calculated initial tumor volume and initial tumor diameter are 9.71 × 10^−8^ cm3 and 57 μm, respectively.

b The tumor's volume was measured at three different time points. The interval between the first and second measurements is denoted as 11_a_. The interval between the second and third measurements is denoted as 11_b_. Each DT treated as a separate case in our analysis.

A review on PubMed using the keywords “medulloblastoma AND (kinetics OR growth rate OR growth pattern OR doubling time OR natural history OR incidentalomas OR incidental)” limited to English articles, up until September 20, 2023, identified six articles that examined the radiological DT of medulloblastoma (Table 2), with reported DTs ranging from 6.84 to 353 days. One of these studies, by Golpayegani et al., 2018 measured tumor volume at three different points, resulting in two distinct DTs, each treated as a separate case in our analysis. With the inclusion of our case, the mean DT across all cases is 53.8 days. Notably, our case exhibited a shorter DT than those previously reported in the literature, highlighting the tumor's aggressive nature.

On the other hand, an article by Zeilhofer et al. (2013) demonstrates the variability in the kinetics that MB can exhibit. He reported a six-year-old boy with an incidental cerebellar mass discovered after minor head trauma, initially presumed to be a low-grade glioma. After two and a half years of dormancy and stable imaging studies, the mass progressed, prompting resection. Histopathological analysis revealed a diagnosis of classical MB.

**Fig. 3:**
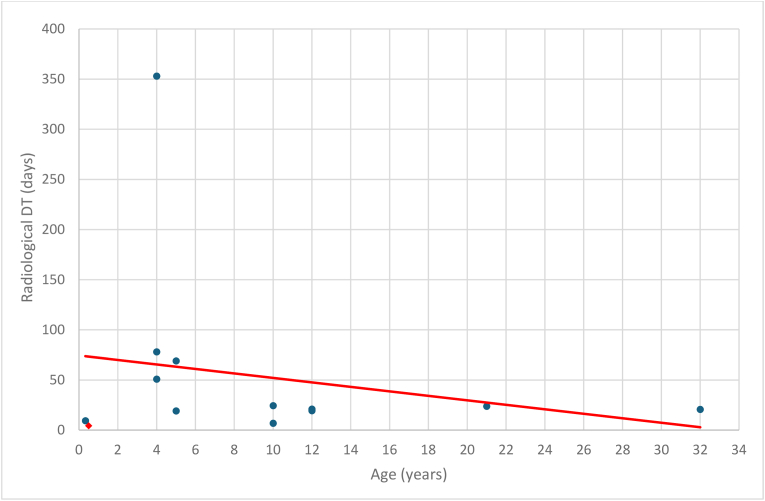
*Regression analysis of patient age and radiological DT across current and reviewed cases.**: The current case DT is represented at 4.3, the average of the calculated range. DT: doubling time.*

Previous studies have shown an inverse relationship between patient age and the histopathological LI of MB. Ito et al. (1992) reported an inverse relationship between patient age and BudR LI. Similarly, Sarkar et al. (2002) reported that pediatric MB exhibit a higher LI and a lower apoptotic index-to-MIB-1 LI ratio compared to adult MB, indicating greater biological aggressiveness of MB in pediatric. In the current study regression analysis of patient age and radiological DT across current and reviewed cases (Fig. 3) revealed a slope of −2.24, indicating an inverse relationship. This suggests that biological aggressiveness increases with age, though the result is statistically nonsignificant (p = 0.48).

Ito et al. also demonstrated that recurrent or metastatic MB exhibited higher LI compared to primary cases, but without satistical significance. Additionaly, a comparison of Ki-67 staining between primary and recurrent tumors revealed differences in proliferation capacity, with higher proliferative indices (>10%) more commonly observed in recurrent MB (Shim et al., 2008). Similarly, our analysis of radiological DT showed a shorter mean DT in recurrent cases (18.4 days) compared to primary cases (64.5 days), suggesting increased growth kinetics in recurrent MB. However, this difference was not statistically significant (p = 0.20), highlighting the need for larger datasets to validate these observations.

Establishing a relationship between histopathological LI and radiological DT was limited by the small number of cases reporting both values and inconsistencies in measurement methods.

Limitations of this study include the assumptions used to calculate the DT, which, although grounded in robust methodologies, inherently carry a degree of uncertainty. Additionally, while the growth rate of a tumor may vary across its components, such as cystic and solid regions, our analysis treated the tumor as a whole. Furthermore, tumor growth rate fluctuations over time, driven by changes in vascularity, oxygen, nutrient supply, and waste elimination (Folkman and Hochberg, 1973), were not accounted for, our study relied on tumor size measurements at only two time points. Constructing a precise and reliable mathematical model of tumor growth requires consideration of these complex and dynamic variables, necessitating further investigations (Murphy et al., 2016).

While the presented case demonstrates the radiological progression of a pediatric MB from non-existence to a symptomatic mass within 42 days, with an exceptionally short DT, a literature review of MBs’ DTs highlights significant variability. This underscores the need for further research into the molecular and genetic factors driving this variability, which could guide the development of more targeted treatments.

## 5 Conclusion

We present the case of a 6-month-old boy who developed obstructive hydrocephalus secondary to a posterior fossa mass 42 days after a negative brain MRI. Histopathological analysis diagnosed the mass as a MB. Kinetic analysis estimated the tumor's DT to be between 2 and 6.5 days, shorter than previously reported cases, indicating a rapidly growing and potentially aggressive tumor. A literature review revealed significant variability in MB DTs, with a mean radiological DT of 53.8 days. Understanding the mutated pathways driving this variability is essential for developing more targeted and effective treatments. These findings highlights the critical importance of prompt diagnosis and intervention in patients with MB.

## Author contributions

(I) Conception and design: Mohammad Y. Hiasat, Yousef M. Odeibat.

(II) Administrative support: Mohammad Y. Hiasat, Mohammad Hazaimeh, Amer A. Alomari.

(III) Provision of study materials or patients: Mohammad Y. Hiasat, Mohammad Hazaimeh, Ala Marji.

(IV) Collection and assembly of data: Mohammad Y. Hiasat, Yousef M. Odeibat, Ala Marji.

(V) Data analysis and interpretation: Yousef M. Odeibat, Ammar S. Al-Omary, Ajwad Obeidat.

(VI) Manuscript writing: Yousef M. Odeibat, Ajwad Obeidat, Amer A. Alomari, Mohammad Y. Hiasat.

(VII) Final approval of manuscript: All authors.

## Ethics statement

The authors are accountable for all aspects of the work in ensuring that questions related to the accuracy or integrity of any part of the work are appropriately investigated and resolved.

The study involves human participants: for this reason, this study conformed to the provisions of the Declaration of Helsinki (1964, as revised in 2013). A written consent for scientific treatment of personal data was obtained from the parents. potentially identifiable human images or data are presented in this study.

## Declaration of generative AI and AI-assisted technologies in the writing process

During the preparation of this work the authors used ChatGPT-4o to enhance manuscript clarity and ensure grammatical correctness. After using this tool/service, the authors reviewed and edited the content as needed and take full responsibility for the content of the publication.

## Funding

This research received no external funding.

## Declaration of competing interest

The authors declare that they have no known competing financial interests or personal relationships that could have appeared to influence the work reported in this paper.
